# Fecal Impaction: An Unusual Cause of Acute Kidney Injury in a Kidney Transplant Recipient

**DOI:** 10.1155/crit/5726025

**Published:** 2025-09-24

**Authors:** Hafsa Tariq, Madhuri Ramakrishnan, Pablo Portocarrero, Mallika Gupta, Nicholas Herrera, Jeffrey Klein, Aditi Gupta, Diane Cibrik

**Affiliations:** ^1^Department of Internal Medicine, University of Rochester, Rochester, New York, USA; ^2^Department of Internal Medicine, The University of Kansas Medical Center, Kansas City, Missouri, USA

## Abstract

Acute kidney injury (AKI) is common in kidney transplant recipients, and the etiology varies depending on the time since transplantation. We present an uncommon case of AKI from obstructive uropathy 7 years posttransplant in a 47-year-old Caucasian male with moderate intellectual disability and end-stage kidney disease secondary to glomerulonephritis who received a deceased donor kidney transplant. He presented with abdominal pain, lethargy, hypercalcemia, and AKI. However, though his serum calcium level improved with intravenous fluid resuscitation, the AKI did not improve. Kidney transplant ultrasound showed hydronephrosis of the transplant ureter, and a noncontrast abdominal and pelvic computed tomography scan showed fecal impaction as the cause of obstruction of the transplanted ureter. The patient underwent fecal disimpaction resulting in the resolution of his hydronephrosis and return of his kidney function to baseline. Although a few case reports have been published of fecal impaction causing AKI due to obstruction of native ureters, to our knowledge, this is the first case describing AKI from fecal impaction in an adult kidney transplant recipient.

## 1. Introduction

Acute kidney injury (AKI) is common after kidney transplant affecting up to 30%–50% of kidney transplant recipients [1]. The differential diagnosis of AKI after kidney transplantation includes rejection, acute tubular necrosis (ATN), recurrent renal disease, BK nephropathy, chronic calcineurin inhibitor exposure, renal artery stenosis, and obstruction. The likelihood of an etiology of AKI in kidney transplant recipients differs based on time posttransplant.

Obstructive uropathy with hydronephrosis is a common cause of AKI post–kidney transplantation [2]. Obstructive hydronephrosis posttransplant often results from allograft ureteral complications and can be due to extrinsic or intrinsic causes. Pelvic fluid collections or masses can cause extrinsic compression of the ureter leading to hydronephrosis. Alternatively, nephrolithiasis, benign prostatic hypertrophy (BPH), vascular insufficiency involving the transplant ureter, rejection, and BK virus infection can cause intrinsic obstruction of the ureter. In addition, urinary bladder dysfunction can lead to nonobstructive hydronephrosis. Lastly, pregnancy can cause hydronephrosis by similar mechanisms.

In native kidneys, fecal impaction has been reported previously as an uncommon cause of obstructive hydronephrosis. It was first reported in 1954 [3], and since then, multiple cases of hydronephrosis in native kidneys due to fecal impaction have been reported [4–15]. Risk factors for fecal impaction include being elderly, neuropsychiatric illness, medications, poor diet and dehydration, and gastrointestinal hypomobility. To our knowledge, hydronephrosis due to fecal impaction has rarely been described in a kidney transplant recipient.

We present a case of an adult kidney transplant recipient who was admitted with abdominal pain, lethargy, hypercalcemia, and AKI 7 years posttransplant and was found to have fecal impaction causing hydronephrosis due to extrinsic obstruction of the ureter. This case is unique due to the paucity of literature in the kidney transplant setting describing fecal impaction as a potential cause of AKI. While several case reports have described this presentation with native kidneys [4–15], we found only one other case report, that too in a pediatric patient, that reported AKI related to fecal impaction in a patient with kidney transplant [16].

## 2. Case Report

A 47-year-old man was admitted to the University of Kansas Medical Center in July 2021 from an assisted living facility for abdominal pain, diarrhea, poor oral intake, and progressive lethargy. He had a history of end-stage kidney disease due to membranoproliferative glomerulonephritis, on dialysis for 11 years prior to transplantation. He underwent a deceased donor kidney transplant in December 2014 with a kidney donor profile index (KDPI) of 68%. He had a calculated panel reactive antibody (cPRA) of 0%, and T- and B-cell crossmatch was negative. The cold ischemic time was 14 h, and he received rabbit antithymocyte globulin for induction. His cytomegalovirus (CMV) status was intermediate risk with both donor and recipient being CMV IgG positive. His posttransplant course was complicated by BK viremia and CMV viremia in 2015–2016, within 1 year posttransplant which were treated successfully. A renal allograft biopsy performed in June 2015 for an episode of AKI showed ATN but no rejection—AKI was eventually concluded to be due to progression to ATN from hypovolemic/prerenal AKI in the setting of diarrhea. His maintenance immunosuppression was tacrolimus (goal trough levels 4–8 ng/mL by HPLC-MS) and prednisone 2.5 mg daily. Mycophenolic acid was discontinued due to the history of BK and CMV viremia. His baseline creatinine fluctuated between 2.0 and 2.5 mg/dL, with estimated glomerular filtration rate (eGFR) fluctuating between 30 and 40 mL/min/1.73 m^2^. Other relevant past medical history included moderate intellectual disability attributed to developmental delay not previously characterized, seizure disorder, chronic malnutrition with body mass index of 14.2 kg/m^2^, chronic diarrhea, recurrent hypomagnesemia, tertiary hyperparathyroidism requiring parathyroidectomy prior to transplantation complicated by recurrent hypocalcemia posttransplant, and osteoporosis.

**Figure 1:**
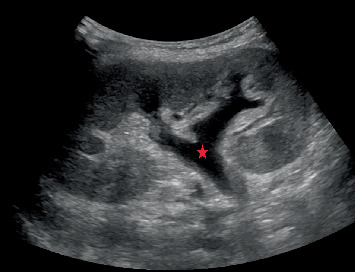
Transplant kidney ultrasound demonstrating hydronephrosis (red star).

**Figure 2:**
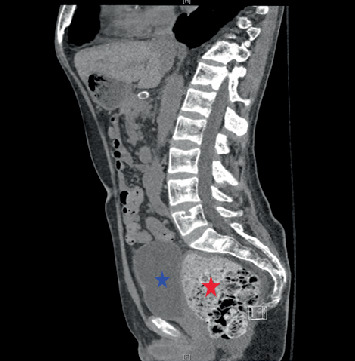
CT scan of the abdomen and pelvis without contrast showing a large fecal impaction (red star) and, in this view, shown to be causing compression of the urinary bladder (blue star).

**Figure 3:**
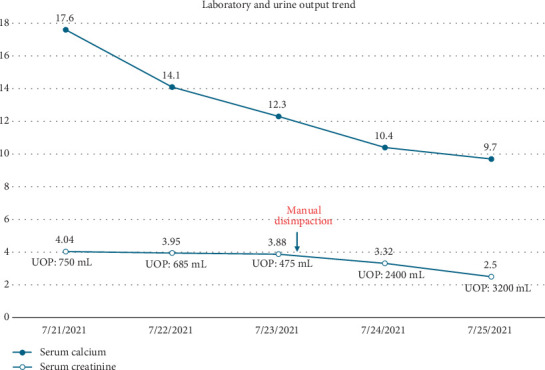
Laboratory data, including serum calcium, serum creatinine, and urine output (UOP) trend during admission.

Upon hospital admission, he was normotensive and afebrile, with oxygen saturation of 100% on room air. On exam, he had dry mucous membranes, diffuse abdominal tenderness, and no peripheral edema. His laboratory work was significant for elevated serum creatinine of 4.04 mg/dL (normal 0.60–1.20 mg/dL; his baseline 2.0–2.5 mg/dL) and serum calcium level of 17.6 mg/dL (normal 8.9–10.3 mg/dL). Urinalysis was bland except for 2–10 white blood cells per high power field and showed no casts or crystals. He was nonoliguric at presentation. His medications at the time of admission included tacrolimus 6 mg twice daily, prednisone 2.5 mg daily, levetiracetam 500 mg twice daily, pantoprazole 40 mg twice daily, teriparatide 20 mcg subcutaneous injection daily, calcitriol 1 mcg twice daily, cholecalciferol 5000 units daily, calcium carbonate 2500 mg four times daily, ferrous sulfate 325 mg twice daily, cyanocobalamin 1000 mcg daily, magnesium oxide 400 mg three times daily, and tramadol 50 mg as needed for pain. His severe hypercalcemia was investigated with parathyroid hormone (PTH) that was appropriately suppressed to < 4 pg/mL (normal 10–65 pg/mL), PTH-related protein (PTHrP) that was not elevated at 0.9 pmol/L (normal ≤ 4.2 pmol/L), vitamin D 25-OH levels that were within a normal limit of 66 ng/mL (normal 30–80 ng/mL), vitamin D 1,25-OH levels that were also within a normal limit of 59 pg/mL (normal 19.9–79.3 pg/mL), and serum protein electrophoresis that showed no monoclonal protein. Hypercalcemia was eventually concluded to be secondary to a combination of profound hypovolemia/intravascular volume depletion and an iatrogenic cause from his high-dose calcium and vitamin D supplementation. AKI was initially presumed secondary to hypovolemia/intravascular volume depletion and hypercalcemia. However, despite aggressive fluid resuscitation and improvement of hypercalcemia, he became oliguric within the subsequent 48 h with tenderness at the allograft site. An ultrasound of the kidney allograft showed hydronephrosis (Figure 1)—the closest previous ultrasound was from 3 months prior that did show mild ectasia of the renal collecting system. However, a noncontrast computed tomography (CT) scan of the abdomen and pelvis showed progression of hydronephrosis to moderate from mild on a CT scan 3 months prior, along with a large amount of retained stool causing fecal impaction with extrinsic compression of the transplanted ureter (Figure 2). After manual stool disimpaction, his oliguria resolved with a rapid return of his kidney function to baseline and normalization of serum calcium level. His laboratory parameters and urine output trend are presented in Table 1 and Figure 3. During the admission, other workup for AKI including BK PCR was negative.

## 3. Discussion

AKI posttransplantation can be due to various etiologies including humoral or cellular rejection, acute tubular injury, recurrent disease, infections including BK nephropathy, vascular complications such as renal artery stenosis, urinary tract obstruction, or medications (chronic exposure to calcineurin inhibitors, etc.) [17]. Hydronephrosis can happen at any time during the transplant course and occurs in up to 10% of kidney transplant recipients [2] and can present clinically as urinary retention, lower abdominal discomfort, and/or impaired kidney function. Hydronephrosis can be due to either intrinsic or extrinsic obstruction of the ureter. In the early posttransplant period, intrinsic obstruction can be due to blood clots within the bladder or from postsurgical edema from ureteroneocystostomy or BPH. Ureteral strictures can also lead to obstructive hydronephrosis and usually present later in the posttransplant period and can be the result of ureteral ischemia, rejection, or infection (related to BK virus). In addition, extrinsic compression of the ureter by pelvic fluid collections or pelvic masses may cause obstructive hydronephrosis. In contrast, nonobstructive hydronephrosis may result from vesicoureteral reflux or bladder atony secondary to diabetes or BPH.

Several cases of fecal impaction have been reported as the cause of hydronephrosis and AKI in native kidneys. Ney and Hyman reported the first case of obstructive uropathy in 1954 caused by fecal impaction in a female patient [3]. Since then, hydronephrosis due to chronic constipation and fecal impaction has been reported intermittently in native kidneys [4–15]. The patients in these cases have had risk factors including being elderly with varying degrees of limitation in mobility and with comorbidities such as dementia, schizophrenia, and chronic constipation. Chronic constipation that is severe enough to impact quality of life, in particular, has been reported in patients with chronic kidney disease (in up to 30%–40% of patients) [18], increasing their risk for fecal impaction. Most of these cases have presented with hydronephrosis and/or bladder compression and AKI of varying degrees of severity. Release of fecaloma through manual disimpaction and enemas resulted in the resolution of hydroureteronephrosis and AKI in many cases. Untreated fecal impaction has, however, led to more serious consequences like spontaneous rupture of the urinary bladder [7]. The previously reported cases are summarized in Table 2.

Kise et al. recently described a case of a 16-year-old kidney transplant recipient with congenital anomalies of the kidney and urinary tract (CAKUT) and anorectal malformation, who had AKI due to a mass of impacted stool in the sigmoid colon and rectum compressing the bladder neck that required manual evacuation of the rectum for improvement in kidney function [16].

Our adult patient did not have any urinary tract or anorectal malformation but did have moderate intellectual disability that likely contributed to a lack of adequate hydration and verbal communication of symptoms. He had other risk factors for the development of fecal impaction including chronic opioids as well as iron supplementation without any reported use of laxatives. With his history of chronic immunosuppression and prior history of diarrhea, his diarrhea on presentation was considered more likely from infectious etiology, and the possibility of overflow diarrhea due to fecal impaction was not explored initially. Hypercalcemia likely contributed to the development of fecal impaction as well. High serum calcium levels decrease gastrointestinal mobility by reducing neuromuscular excitability which leads to atonia of gastric smooth muscles [19]. Individuals with chronic kidney disease often have dysregulated calcium homeostasis which leads to decreased capacity of calcium buffering [20].

## 4. Conclusion

In summary, we report a case of AKI in an adult kidney transplant recipient related to an uncommon cause—fecal impaction causing extrinsic obstructive hydronephrosis. Though described in several case reports in the setting of native kidneys, it remains a rare cause of AKI. To our knowledge, this is the first case describing fecal impaction as a potential cause of AKI in an adult with a kidney transplant, although the resolution of AKI was likely due to a combination of both the relief of obstruction and improvement in hypercalcemia. Though infrequent, fecal impaction should be considered in the differential diagnosis for AKI in patients with multiple risk factors. Prompt imaging is required to make the diagnosis, which can lead to timely treatment and recovery. Attention must also be paid to prevention strategies particularly in the elderly and those with chronic kidney disease who are at risk for chronic constipation, including active emphasis on a fiber-rich diet and mobility, periodic review of the medication list to discontinue/limit contributing medications, and periodic review of symptoms with appropriate use of laxatives [21].

## Figures and Tables

**Table 1:** Laboratory values on presentation and subsequent days.

**Laboratory parameters**	**On presentation**	**After 48 h**	**After 96 h**
Hemoglobin (g/dL)	10.0		
White blood cell count (/*μ*L)	8.4		
Platelets (/*μ*L)	194		
Sodium (mEq/L)	136	140	143
Potassium (mmol/L)	3.3	3.2	3.8
Chloride (mEq/L)	96	101	108
Bicarbonate (mEq/L)	34	29	26
Blood urea nitrogen (mg/dL)	88	60	30
Creatinine (mg/dL)	4.04	3.88	2.5
Glucose (mg/dL)	103	126	138
Albumin (g/dL)	3.4		
Calcium (mg/dL)	17.6	12.3	9.7
Tacrolimus (ng/mL)	9		
Parathyroid hormone (PTH) (pg/mL)	< 4		
PTH-related peptide (pmol/L)	0.9		
25-OH vitamin D (ng/mL)	66.6		
1,25-OH vitamin D (pg/mL)	59.0		

**Table 2:** Prior case reports of hydronephrosis/AKI in the setting of fecal impaction.

**Paper**	**Year**	**Journal**	**Article type**	**Patient demographics**	**Important comorbidities**	**Presentation**	**Management**	**Follow-up**
Claffey et al. [4]	1995	Am Surg	Case report	Adult female	—	Bilateral hydronephrosis	Ureteral stents and manual disimpaction	—
Knobel et al. [5]	2007	J Clin Gastroenterol	Case report	81-year-old female	Dementia (nursing home resident)	Bilateral hydronephrosis Serum creatinine: 0.9	Rectosigmoid lavage and manual disimpaction	Repeat ultrasound with resolution of hydronephrosis
Tan et al. [6]	2008	Kidney Int	Case report	71-year-old male	Diabetes mellitus	Displaced urinary bladder and right hydronephrosis	Manual disimpaction	Repeat ultrasound with resolution of hydronephrosis
Chute et al. [7]	2009	Am J Forensic Med Pathol	Case report	47-year-old female	Schizophrenia	Bladder perforation and large bowel distension with fecaloma	(Patient death due to bladder perforation)	(Patient death due to bladder perforation)
Gonzalez [8]	2010	Am J Hosp Palliat Care	Case reports (2)	75-year-old female	Dementia (nursing home resident)	Moderate right hydronephrosis AKI with serum creatinine 1.4 mg/dL	IV fluids and enemas	Return of creatinine to baseline
				88-year-old female	Dementia (nursing home resident)	Right hydronephrosis AKI with serum creatinine 3.9 mg/dL	IV fluids, vasopressors, eventually hospice	—
Perfecto Valero et al. [9]	2019	Cir Esp (Engl Ed)	Case report	90-year-old female	Chronic constipation	Right hydronephrosis AKI with serum creatinine 3.23 mg/dL	Manual disimpaction	Improvement in serum creatinine
Khan et al. [10]	2019	JGH Open	Case report	48-year-old female	Schizophrenia	Bilateral hydronephrosis AKI with serum creatinine 2.37 mg/dL	Laparotomy with sigmoid colon resection and end colostomy	—
Joo and Lee [11]	2020	Ann Geriatr Med Res	Case report	83-year-old female	Dementia, chronic constipation, bed-ridden	Right hydronephrosis “Mildly increased” serum creatinine at 0.93 mg/dL	Enemas and manual disimpaction	Repeat ultrasound with resolution of hydronephrosis and serum creatinine 0.69 mg/dL
Ozlu et al. [12]	2020	Cureus	Case report	88-year-old female	Diabetes mellitus	Bilateral hydronephrosis AKI with serum creatinine 3.49 mg/dL	Rectosigmoid lavage and manual disimpaction	Repeat CT scan with improvement in hydronephrosis and serum creatinine 1.37 mg/dL
Kise et al. [16]	2021	Indian Journal of Transplant	Case report	16-year-old female	Kidney transplant, CAKUT, and anorectal malformation	Hydronephrosis of transplant ureter AKI with serum creatinine 0.85 mg/dL	Lavage and manual disimpaction	Serum creatinine 0.45 mg/dL Ultrasound 2 months later with improved hydronephrosis
Mchirgui et al. [13]	2021	F1000Res	Case report	74-year-old female	Dementia, chronic constipation	Bladder compression	Enemas and manual disimpaction	(Patient death from septic shock)
Almazan and Petersen [14]	2025	ACG Case Rep J	Case report	86-year-old female	Cerebral amyloid angiopathy	Mild bilateral hydronephrosis AKI with serum creatinine 1.30 mg/dL (baseline 0.90 mg/dL)	Manual disimpaction under anesthesia	“Resolution of obstruction and AKI”
Al Jardali et al. [15]	2025	Ann Med Surg (Lond)	Case report	76-year-old female	Immobility/bed-ridden	Bilateral hydronephrosis AKI with serum creatinine 1.70 mg/dL	Manual disimpaction and fleet enemas	Repeat CT scan with complete resolution of hydronephrosis and serum creatinine 0.46 mg/dL

## Data Availability

Research data are not shared. The data presented in this case report is not publicly available due to privacy concerns related to patient data.
